# Pregnancy and postpartum experiences of elite para athletes: a cross-sectional survey

**DOI:** 10.3389/fspor.2026.1904745

**Published:** 2026-08-12

**Authors:** Erica H. Gavel-Pinos, Tara-Leigh McHugh, Margie H. Davenport

**Affiliations:** 1 Program for Pregnancy and Postpartum Health, Physical Activity and Diabetes Laboratory, Faculty of Kinesiology, Sport and Recreation, Women and Children’s Health Research Institute, Alberta Diabetes Institute, University of Alberta, Edmonton, AB, Canada; 2 School of Kinesiology, University of Calgary, Calgary, AB, Canada

**Keywords:** health, para athlete, postpartum, pregnancy, training

## Abstract

**Background:**

Pregnancy and the postpartum period present unique physiological, health, and sport-related challenges for female Para athletes, yet little is known about their experiences during these life stages. This study aimed to describe demographic, reproductive, health, and sport characteristics, and to examine training practices, access to guidance, and return-to-sport experiences among elite Para athletes who experienced pregnancy.

**Methods:**

A cross-sectional survey was completed by 25 current or former elite Para athletes who had experienced pregnancy while participating in high-performance sport. Participants completed an anonymous online questionnaire assessing demographics, disability characteristics, pregnancy and postpartum health outcomes, delivery outcomes, sport participation, training modifications, and return-to-sport timelines. Descriptive statistics were used to summarize outcomes.

**Results:**

Participants were 36 ± 6 years of age and represented a range of impairments and sport types. Commonly reported health conditions included urinary incontinence (20%), anxiety (16%), low back pain (16%), and depression (12%). Pregnancy and neonatal outcomes were generally favorable, with a median gestational age of 38 weeks (IQR: 2), mean birth weight of 7.1 ± 1.1 lbs, and a 4% neonatal intensive care unit admission rate. Less than half of participants (43%) reported receiving advice regarding physical activity during pregnancy, despite 57% modifying their training and 39% reporting exposure to higher-risk training conditions. Median return-to-exercise, training, and competition occurred at 6, 14, and 20 weeks postpartum, respectively.

**Conclusions:**

Female Para athletes generally reported favorable pregnancy and neonatal outcomes while maintaining sport participation. However, substantial gaps in pregnancy-specific and disability-informed guidance were identified. These findings highlight the need for tailored clinical recommendations, integrated support systems, and structured return-to-sport pathways to optimize health and performance during pregnancy and postpartum in Para sport.

## Introduction

Engagement in sport can be a contributor to physical health, functional capacity, and overall quality of life among women with disabilities. However, pregnancy and the postpartum period introduce complex physiological, functional, and sport-related challenges that may uniquely interact with impairment type, training demands, and competitive context (1–3). In addition to well-described changes in cardiovascular, endocrine, and musculoskeletal systems, female Para athletes must often navigate decisions related to training modification, safety, and return-to-sport in the absence of clear, impairment-specific guidance (4, 5).

During pregnancy and postpartum, female Para athletes may experience changes across multiple health domains including mental health, endocrine and metabolic function, pelvic health, bladder and bowel function, and sleep. Yet these health outcomes remain underexplored in the literature. Emerging evidence in both general and Para populations suggests that pregnancy-related conditions such as gestational diabetes, hypertensive disorders, pelvic girdle pain, and urinary incontinence are relatively common and may persist into the postpartum period, influencing quality of life and return-to-sport trajectories (6–8). In athletes with disabilities, these challenges may be further shaped by impairment-specific considerations, including neurogenic bladder dysfunction, altered autonomic regulation, and differences in mechanical loading, which may influence both symptom presentation and management (9–11). Concurrent mental health conditions, including anxiety and depression, add further complexity to postpartum recovery and sport reintegration (12).

Beyond health outcomes, there is a critical need to better understand how female Para athletes engage in training during pregnancy, the extent to which they receive evidence-informed advice, and how they navigate return-to-sport. Current evidence in the able-bodied population highlights variability in guidance, training practices, and timelines for return-to-exercise and competition, often influenced by access to knowledgeable healthcare providers and sport systems (13–15). However, these considerations are largely unexplored in Para sport, where athletes may face additional barriers related to accessibility, classification demands, and impairment-specific risk profiles (16, 17). The absence of tailored guidance may contribute to uncertainty around training safety, exposure to higher-risk conditions, and inconsistent return-to-sport pathways.

Therefore, the purpose of this study was to (1) characterize the demographic, reproductive, and sport characteristics of elite female Para athletes with pregnancy experience, (2) describe the prevalence of pregnancy- and postpartum-related health conditions and birth outcomes, and (3) examine training practices and return-to-sport experiences. Specifically, this study aimed to describe health across multiple domains during pregnancy and postpartum while documenting athletes’ experiences with training, competition, and return-to-sport.

## Materials and methods

### Study design and participants

This cross-sectional survey study examined demographic, reproductive, health, and sport-related experiences among elite Para athletes during pregnancy and the postpartum period, with a particular focus on health outcomes, training practices, access to guidance, and return-to-sport experiences. Athletes were eligible if they were aged ≥18 years and had experienced pregnancy while participating in elite or high-performance sport. Participants were recruited between June 2025 and January 2026 through Paralympic and Para sport networks, national sport organizations, athlete commissions, and social media. Electronic informed consent was obtained prior to participation. The study was approved by the University of Alberta Research Ethics Board (Pro00151952) and conducted in accordance with the Declaration of Helsinki.

### Survey development

The questionnaire consisted of approximately 85 primary items and was developed by a multidisciplinary research team with expertise in Para sport, pregnancy, and athlete health. Where available, questionnaire items were adapted from established surveillance instruments, standardized terminology, and evidence-based guidelines, including the Pregnancy Risk Assessment Monitoring System (PRAMS) for maternal and pregnancy-related health measures (18), the International Continence Society (ICS)/International Urogynecological Association (IUGA) terminology for obstetric pelvic floor disorders (19), the International Paralympic Committee (IPC) Athlete Classification Code for impairment and classification-related items (20), the 2019 Canadian Guideline for Physical Activity throughout Pregnancy for physical activity and exercise-related questions (26), and the 2023 International Olympic Committee (IOC) Consensus Statement on Relative Energy Deficiency in Sport (REDs) for athlete health-related content (21). Additional questions specific to Para sport, pregnancy, postpartum experiences, and elite athlete participation were developed by the multidisciplinary research team to address aspects not captured by existing instruments. The survey was designed to capture demographic, reproductive, disability-related, and sport characteristics, as well as pregnancy and postpartum health outcomes, training practices, access to physical activity guidance, and return-to-sport experiences among female Para athletes. Prior to distribution, the questionnaire was piloted by members of the research team and individuals familiar with the target population to evaluate the clarity, usability, survey flow, and technical functionality of the online platform. Based on feedback from pilot testing, minor revisions were made to improve question wording, response options, survey flow, and overall user experience before the questionnaire was finalized and distributed.

### Data collection

Data was collected via an anonymous, self-administered online questionnaire hosted on REDCap (Research Electronic Data Capture). The survey (20–30 min) included fixed-response, multiple-response, Likert-scale, and optional open-text items, with responses editable prior to submission and stored securely. Measures included demographics (age, country of birth), reproductive characteristics (pregnancy status, time since pregnancy, number of children), sport type (endurance, team, strength/power, skill/precision), and training characteristics during pregnancy and postpartum. Health domains included questions surrounding mental health, endocrine/metabolic, pelvic health, bladder/bowel function, female athlete health (i.e., relative energy deficiency syndrome), and sleep, with outcomes assessed during pregnancy and postpartum. Pregnancy and delivery outcomes included delivery mode, gestational age, birth weight, Neonatal Intensive Care Unit (NICU) admission, perineal tearing, and delivery complications, as well as breastfeeding status and duration. Given the highly specialized nature of the study population, a target sample size was not predetermined. Instead, all individuals who expressed interest following extensive recruitment efforts and met the inclusion criteria were invited to participate, with recruitment concluding when no additional eligible participants expressed interest during the recruitment period.

### Statistical analysis

Statistical analyses were performed using SPSS (IBM Corp., Armonk, NY, USA). Continuous variables are presented as mean ± standard deviation (SD) or median [interquartile range (IQR)] based on distribution. Categorical variables are reported as frequencies and percentages. Descriptive analyses were used to summarize demographic, clinical, and sport characteristics. Paired pregnancy and postpartum outcomes were compared descriptively. Given the descriptive objectives of the study, the relatively small sample size, and the highly specialized study population, inferential statistical analyses were not performed. Accordingly, the findings are presented descriptively and should be interpreted as exploratory.

## Results

Twenty-five participants were included (age: 36 ± 6 years; Table 1). Participants were born across multiple countries, most commonly Canada (36%), followed by China and the United Kingdom (12% each), with all other countries represented by ≤8%. Participants reported a range of disability types, most commonly amputation and neurological conditions (32% each), followed by spinal cord injury (24%), other conditions (16%), and visual impairment (12%), with some participants reporting more than one disability; therefore, percentages exceed 100% as participants could report multiple disabilities/impairments. The majority of participants were postpartum (92%), with 8% currently pregnant. Median time since most recent pregnancy was 36 months (IQR: 86), and participants reported a median of one child (IQR: 1). Sport participation was primarily in endurance (36.8%) and team sports (36.8%), with additional representation in skill/precision sports (26.3%); no participants reported strength/power sports (Table 1).

**Table 1:** Participant demographic, disability, reproductive, and sport characteristics.

Variable		Total sample (*n* = 25)
Age (years), mean ± SD		36 ± 6
Country of birth, *n* (%)		
	Canada	4 (36.0)
	China	3 (12.0)
	Malaysia	1 (4.0)
	Netherlands	2 (8.0)
	Nicaragua	1 (4.0)
	Niger	1 (4.0)
	Pakistan	1 (4.0)
	Slovenia	1 (4.0)
	Uganda	2 (8.0)
	United Kingdom	3 (12.0)
Type of disability/impairment, *n* (%)		
	Spinal cord injury	6 (24.0)
	Amputation	8 (32.0)
	Neurological	8 (32.0)
	Visual impairment	3 (12.0)
	Other	4 (16.0)
Pregnancy status, *n* (%)		
	Currently pregnant	2 (8.0)
	Postpartum	23 (92.0)
	Time since most recent pregnancy (months), median (IQR)	36 (86)
	Number of children, median (IQR)	1 (1)
Sport type, *n* (%)		
	Endurance	7 (36.8)
	Team sport	7 (36.8)
	Strength/power	0 (0.0)
	Skill/precision	5 (26.3)

SD, standard deviation; IQR, interquartile range. Data are presented as *n* (%) unless otherwise indicated. Continuous variables are presented as mean ± SD or median (IQR), as appropriate. Participants could report more than one disability/impairment; therefore, percentages for disability type may exceed 100%. Sport types were categorized according to the primary physiological demands of participants’ primary sport.

Health conditions were reported across multiple domains (Table 2). Anxiety (16%) and depression (12%) were the most commonly reported mental health conditions. Endocrine/metabolic conditions included diabetes (12%), thyroid disease (4%), and polycystic ovary syndrome (4%). Pelvic health concerns included low back pain (16%), pelvic girdle pain (12%), and pelvic organ prolapse (8%). Urinary incontinence was reported by 20% of participants, while fecal and wind incontinence were each reported by 8%. No participants reported relative energy deficiency syndrome (REDs). Sleep-related conditions included insomnia (12%) and sleep apnea (8%).

**Table 2:** Self-reported health conditions among elite female para athletes.

Health condition		Total sample (*n* = 25)
Mental health, *n* (%)
	Anxiety	4 (16.0)
	Depression	3 (12.0)
Endocrine/metabolic, *n* (%)
	Thyroid disease	1 (4.0)
	Diabetes (Type 1 or Type 2)	3 (12.0)
	PCOS	1 (4.0)
Pelvic health, *n* (%)
	Pelvic girdle pain during pregnancy	3 (12.0)
	Low back pain during pregnancy	4 (16.0)
	Pelvic organ prolapse	2 (8.0)
Bladder/Bowel, *n* (%)
	Urinary incontinence	5 (20.0)
	Fecal incontinence	2 (8.0)
	Wind incontinence	2 (8.0)
Female athlete health, *n* (%)
	Relative Energy Deficiency	0 (0.0)
Sleep, *n* (%)
	Insomnia	3 (12.0)
	Sleep apnea	2 (8.0)

PCOS, polycystic ovary syndrome. Data are presented as *n* (%). Participants were permitted to report multiple health conditions; therefore, percentages may exceed 100%.

When examining health outcomes across pregnancy and postpartum, several conditions persisted or declined over time (Table 3). Anxiety prevalence remained stable (16%), whereas depression differed during pregnancy and postpartum, with 12% during pregnancy and 4% postpartum. Similarly, urinary incontinence differed during the pregnancy and postpartum periods, 16% during pregnancy and 12% postpartum. Pelvic health outcomes, including pelvic girdle pain, low back pain, and pelvic organ prolapse, were reported less frequently postpartum.

**Table 3:** Pregnancy and postpartum health conditions.

Health outcome		During pregnancy (*n* = 23)	Postpartum (*n* = 23)
Mental health, *n* (%)
	Anxiety	4 (16)	4 (16)
	Depression	3 (12)	1 (4)
Endocrine/metabolic, *n* (%)
	Gestational diabetes	3 (12)	1 (4)
	Gestational hypertension	2 (8)	1 (4)
	Preeclampsia	1 (4)	0 (0)
	Hyperemesis gravidarum	1 (4)	0 (0)
Pelvic health, *n* (%)
	Pelvic girdle pain	3 (12)	2 (8)
	Low back pain	3 (12)	2 (8)
	Diastasis recti	2 (8)	1 (4)
	Pelvic organ prolapse	2 (8)	1 (4)
Bladder/bowel, *n* (%)
	Urinary incontinence	4 (16)	3 (12)
	Fecal incontinence	2 (8)	1 (4)
	Wind incontinence	2 (8)	1 (4)
Sleep, *n* (%)
	Sleep apnea	1 (4)	1 (4)
	Insomnia	3 (12)	2 (8)

Data are presented as *n* (%). Participants could report more than one health condition; therefore, percentages within each time period do not sum to 100%.

Regarding pregnancy and delivery outcomes (Table 4), vaginal unassisted delivery was the most common mode (32%), followed by emergency caesarean section (16%) and planned caesarean section (12%). No participants reported perineal tearing. Median gestational age at delivery was 38 weeks (IQR: 2), and mean birth weight was 7.1 ± 1.1 lbs. NICU admission occurred in 4% of cases. Postpartum hemorrhage was reported by 8% of participants, with no cases of shoulder dystocia. Breastfeeding was reported by 57% of participants, with a median duration of 6 months (IQR: 4.5).

**Table 4:** Pregnancy, delivery, and return-to-sport outcomes among elite female para athletes.

Outcome		Total sample (*n* = 23)
Delivery mode, *n* (%)		
	Vaginal, unassisted	8 (32)
	Vaginal, assisted	0 (0)
	Planned caesarean section	3 (12)
	Emergency caesarean section	4 (16)
Perineal tearing, *n* (%)		
	Yes	0 (0)
	No	25 (100)
Gestational age at delivery (weeks), median (IQR)		38 (2)
Birth weight (lbs), mean ± SD		7.1 ± 1.1
NICU admission, *n* (%)		
	Yes	1 (4)
	No	22 (96)
Delivery complications, *n* (%)		
	Postpartum hemorrhage	2 (8)
	Shoulder dystocia	0 (0)
Breastfeeding, *n* (%)		
	Yes	13 (57)
	No	10 (43)
Duration of breastfeeding (months), Median (IQR)		6 (4.5)
Received advice about physical activity during pregnancy, *n* (%)		
	Yes	10 (43)
	No	13 (57)
Source of Advice, *n* (%)		
	Healthcare provider	14 (61)
	Coach	4 (17)
	Family/friends	3 (13)
	Internet/resources	10 (43)
Duration maintaining pre-pregnancy training (weeks), median (IQR)		16 (20)
Modified training during pregnancy, *n* (%)		
	Yes	13 (57)
	No	10 (43)
Exposure to higher-risk training conditions, *n* (%)		
	Yes	9 (39)
	No	14 (61)
Falls during physical activity with concern for fetal health, *n* (%)		
	Yes	1 (9)
	No	21 (91)
Sport-related injury during pregnancy, *n* (%)		
	Yes	0 ()
	No	23 (100)
Return-to-exercise postpartum (weeks), median (IQR)		6 (6)
Return-to-high-impact activity (weeks), median (IQR)		14 (22)
Return-to-training (weeks), median (IQR)		14 (20)
Return-to-competition (weeks), median (IQR)		20 (16)
Sport-related injury postpartum, *n* (%)		
	Yes	2 (9)
	No	21 (91)

SD, standard deviation; IQR, interquartile range; NICU, neonatal intensive care unit. Data are presented as *n* (%) unless otherwise indicated. Continuous variables are presented as mean ± SD or median (IQR), as appropriate. Participants could report more than one source of physical activity advice and more than one delivery complication; therefore, percentages for these variables may exceed 100%.

Sport participation and training behaviors during pregnancy and postpartum are presented in Table 4. Less than half of participants (43%) reported receiving advice about physical activity during pregnancy, while 57% did not receive any guidance. Among those who did receive advice, sources included healthcare providers (61%), internet/resources (43%), coaches (17%), and family or friends (13%). Participants maintained pre-pregnancy training for a median of 16 weeks (IQR: 20), with 57% reporting that they modified their training during pregnancy. Notably, 39% reported exposure to higher-risk training conditions, and 9% experienced falls during physical activity that raised concern for fetal health; however, no sport-related adverse outcomes or injuries were reported during pregnancy.

Postpartum return-to-activity timelines indicated a relatively early resumption of exercise, with a median return at 6 weeks (IQR: 6). Return-to-higher-impact activity and structured training both occurred at a median of 14 weeks (IQR: 22 and 20, respectively), while return to competition occurred later at a median of 20 weeks (IQR: 16). Sport-related injury postpartum was reported by 9% of participants.

## Discussion

This study provides novel insight into the health, pregnancy outcomes, and sport participation experiences of female Para athletes. Overall, the findings suggest that pregnancy-related health and neonatal outcomes are generally favorable and comparable to those reported in athletic populations (3, 15), but important gaps exist in access to guidance, training practices, and return-to-sport pathways.

Participants reported health concerns across multiple domains, including mental health, pelvic health, and bladder/bowel function. Urinary incontinence, low back pain, and anxiety were among the most commonly reported conditions, consistent with findings in general populations (22–24). Depression and urinary incontinence were reported less frequently at postpartum time points than during pregnancy, whereas anxiety remained commonly reported across pregnancy and postpartum, suggesting that mental health concerns may warrant continued attention beyond the immediate postpartum period (12). These findings highlight the importance of multidisciplinary care that extends into the postpartum phase and considers impairment-specific factors, such as neurogenic bladder dysfunction and altered autonomic regulation, which may influence symptom presentation and management (25–27).

Pregnancy and neonatal outcomes were generally favorable, with gestational age, birth weight, and complication rates within expected ranges (28, 29). These findings support the feasibility of pregnancy among female Para athletes when appropriately supported and are consistent with evidence demonstrating the safety of prenatal exercise (3, 15, 30). However, the heterogeneity of impairments reinforces the need for individualized clinical care tailored to both obstetric and sport-specific considerations (27).

A key contribution of this study is the characterization of training behaviors and return-to-sport experiences. Less than half of participants reported receiving advice about physical activity during pregnancy, indicating a substantial gap in access to guidance despite established clinical recommendations (30). Among those who sought information, healthcare providers, and internet-based resources were the most commonly reported sources. The prominence of internet resources is notable given the limited availability of disability-specific evidence and guidance related to pregnancy in Para sport. This finding suggests that many athletes may be relying on information that is not tailored to their impairment, sport demands, or performance goals. Despite this, many participants continued training during pregnancy, often modifying their routines, and a notable proportion reported exposure to higher-risk training conditions. Collectively, these findings suggest that athletes are frequently navigating training and return-to-sport decisions with limited access to consistent, evidence-informed, and Para-specific support, a challenge that has also been identified in broader athlete pregnancy literature (15).

Postpartum return-to-sport timelines indicated early resumption of activity, with return-to-exercise at approximately 6 weeks and progression to higher-impact activity and training around 14 weeks, followed by return to competition at approximately 20 weeks. While broadly consistent with abled-bodied athletic populations, the variability observed highlights the individualized nature of recovery (3, 32, 33). The occurrence of postpartum injury further underscores the need for structured, staged return-to-sport approaches that integrate physical recovery, pelvic health, and sport-specific demands.

Collectively, these findings highlight a critical gap in disability-specific education, screening, and clinical guidance related to pregnancy and sport participation. Improved integration of Para sport considerations into clinical care, alongside stronger collaboration between healthcare providers and sport systems, is needed to better support athletes during this life stage (31, 34, 35).

### Limitations

The relatively small sample size limits generalizability and may not capture the full range of experiences across different impairments and sports. Furthermore, the rarity of this population and the resulting sample size precluded meaningful subgroup analyses by impairment type or sport, highlighting the need for larger, multicenter studies to better understand impairment-specific experiences and outcomes. The cross-sectional, retrospective design introduces potential recall bias, particularly for participants reporting on past pregnancies. The heterogeneity of the sample may obscure impairment-specific patterns, and reliance on self-reported data may introduce reporting bias, particularly for sensitive outcomes such as pelvic and mental health. The absence of a comparator group also limits direct comparison with non-disabled athlete populations.

### Future research directions

Future research should prioritize prospective, longitudinal designs to better understand health, training, and return-to-sport trajectories across pregnancy and postpartum in female Para athletes. Larger, impairment-specific cohorts are needed to examine how different disability types influence outcomes. Moreover, intervention-based research is also warranted to develop and evaluate tailored education, clinician-guided training recommendations, and structured return-to-sport protocols. Incorporating athlete-centered and co-design approaches will be critical to ensure that future strategies are practical and responsive to the needs of female Para athletes. In addition, future studies should evaluate the implementation and effectiveness of disability-specific clinical guidelines and multidisciplinary models of care across diverse Para sport settings. Establishing international collaborations and standardized data collection will also be essential to improve sample sizes, facilitate comparisons across impairment groups and sports, and strengthen the evidence base supporting pregnancy and postpartum care in female Para athletes.

## Conclusion

In this cross-sectional survey of elite female Para athletes, pregnancy and neonatal outcomes were generally favorable, supporting the feasibility of pregnancy alongside high-performance sport. However, athletes reported a broad range of health conditions during pregnancy and postpartum, with several remaining prevalent beyond delivery highlighting the need for ongoing, multidisciplinary care. Importantly, substantial gaps were identified in access to physical activity guidance, with many athletes continuing to train during pregnancy despite limited disability-specific support and exposure to potentially higher-risk training conditions.

Return-to-sport experiences were characterized by early but highly variable resumption of training and competition, underscoring the individualized nature of postpartum recovery and the need for structured, impairment-informed return-to-sport pathways. Collectively, these findings reveal a critical gap between the participation of female Para athletes in high-performance sport and the availability of evidence-based clinical guidance to support them throughout pregnancy and postpartum. Developing impairment-specific recommendations, integrated models of care, and evidence-informed training guidelines should be a priority to optimize maternal health, athletic performance, and safe return to sport for this growing and historically underserved athlete population.

## Data Availability

The original contributions presented in the study are included in the article/Supplementary Material, further inquiries can be directed to the corresponding author.
