# Supplementary material for: Pregnancy and postpartum experiences of elite para athletes: a cross-sectional survey
Source: Front Sports Act Living. 2026 Aug 12;8:1904745. doi: 10.3389/fspor.2026.1904745 (PMC13506788; doi:10.3389/fspor.2026.1904745)
Supplement: Supplementary file 1 [file Datasheet1.pdf]

Experiences of prenatal care and physical activity among pregnant and postpartum Para-athletes.

This survey is interested in the experiences of pregnancy among Para athletes who compete and train at the elite and Paralympic level. You will be asked questions surrounding your health, prenatal care experiences, training, and competition, before, during, and after pregnancy.

Eligibility Questions:

The following questions will determine your eligibility for the present study.

1. Did you become pregnant while as an elite athlete?
    a. Yes
    b. Yes, I am currently pregnant and training
    c. No
2. What is the highest level you competed at during of following pregnancy?
    a. Paralympic Games
    b. World championship
    c. Collegiate/University Level
    d. None of the above

Demographics:

1. What is your age? (DROPDOWN)

2. What was your sex at birth? (RADIO)
    a. Female
    b. Male

3. What is your gender identity? Please select all that apply.
    a. Woman
    b. Man
    c. Non-binary
    d. Other
        i. Please describe
    e. Prefer not to say

4. What is your ethnicity?
    a. Asian or Pacific Islander
    b. Black
    c. Hispanic or Latino/a/x
    d. Indigenous (e.g., North American Indian Navajo, South American Indian Quechua, Aboriginal, or Torres Strait Islander
    e. Middle Eastern or North African
    f. White
    g. Prefer to self-identify (OPEN BOX)

h. Prefer not to answer

5. What is your country of birth?
    a. Drop down list of countries

6. What is your highest level of education?
    a. Primary education (e.g. elementary education, grades 1-6)
    b. Lower secondary education (e.g. junior secondary school, middle school, junior high school, grades 7-9)
    c. Upper secondary education (e.g. secondary school, high school, senior high school, grades 10-13)
    d. Post secondary education
        i. Trade certificate, technician diploma, professional certification
        ii. Bachelor's degree
        iii. Masters' degree
        iv. Doctoral degree
        v. Professional Degree

7. What physical disability are you diagnosed with? Select all that apply.
    a. Multiple Sclerosis
    b. Spinal Cord Injury
        i. Paraplegia
        ii. Tetraplegia/Quadriplegia
    c. Scoliosis
    d. Epilepsy
    e. Spina Bifida
    f. Blindness
    g. Loss of Hearing or deaf
    h. Traumatic Brain Injury
    i. Cerebral Palsy
    j. Cystic Fibrosis
    k. Muscular Dystrophy
    l. Tourette Syndrome
    m. Dwarfism
    n. Arthritis
    o. Rheumatoid Arthritis
    p. Limb Difference
    q. Prader-Willi Syndrome
    r. Hashimoto's Disease
    s. Poliomyelitis
    t. Hansen's Disease
    u. Chronic pain (not due to an impairment diagnosis)
    v. Other – Please describe

8. Do you use a mobility device?
    a. Yes, all of the time
    b. Yes, most of the time

c. Yes, some of the time
d. Yes, very rarely
e. Never

9. What is your classification profile?
    a. Impaired muscle power
    b. Impaired passive range of movement
    c. Limb deficiency
    d. Leg length difference
    e. Short stature
    f. Hypertonia
    g. Ataxia
    h. Athetosis
    i. Vision impairment
    j. Intellectual impairment

10. How long since the end of your most recent pregnancy
    a. I am currently pregnant
        i. How many weeks pregnant (DROPDOWN)
    b. I am postpartum
        i. How many months/years postpartum (DROPDOWN)

11. How many children (of any age) do you have? (DROP DOWN)
    a. (ASKED FOR EACH CHILD)

12. What is your height
    a. Answer in ft, inches
        i. DROP DOWN
    b. Answer in cm
        i. OPEN BOX NUMBER VALIDATION

Sport History

1. What sports do you compete in?
    a. Para alpine skiing
    b. Para archery
    c. Para athletics
    d. Para badminton
    e. Para biathlon
    f. Blind football
    g. Boccia
    h. Para canoe
    i. Para cross-country skiing
    j. Para cycling
    k. Para equestrian
    l. Goalball
    m. Para ice hockey
    n. Para judo

o. Para powerlifting
p. Para rowing
q. Shooting para sport
r. Sitting volleyball
s. Para snowboard
t. Para swimming
u. Para table tennis
v. Para taekwondo
w. Para triathlon
x. Wheelchair basketball
y. Wheelchair curling
z. Wheelchair fencing
aa. Wheelchair rugby
bb. Wheelchair tennis
cc. Other
    i. Open box

Supports and barriers to competing as a Para athlete while pregnant or as a parent:

The following questions will ask about supports and barriers related to pregnancy or parenthood that you have experienced while competing at the Olympic or Paralympic Games.

1) What is <u>being done well to support</u> pregnant Para athletes by the IPC, your International Federation or your National Sport Organization?
    a. Open box
2) What do you think <u>can be done to improve</u> the overall support for pregnant Para athletes by the IPC, your International Federation or your National Sport Organization?
    a. Open box
4) What do you think is <u>currently being done well to</u> <u>support</u> postpartum/parenting Para athletes by the IPC, your International Federation or your National Sport Organization?
    a. Open box
3) What do you think <u>can be done to improve the overall support</u> for postpartum/parenting Para athletes by the IPC, your International Federation or your National Sport Organization?
    b. Open box

Health Outcomes Prior to Pregnancy

1. BEFORE your pregnancy or becoming a parent, did you experience any of the following medical conditions or symptoms? Check all that apply.
    a. No medical conditions or symptoms
    b. Eating Disorder
    c. Thyroid Disease
    d. Type 1 Diabetes
    e. Type 2 Diabetes
    f. Anxiety
    g. Depression
    h. Anemia

i. Pelvic girdle pain (pain in the back or front of the pelvis/groin and/or hips, either on one side or both)
j. Low back pain
    i. With sciatica
    ii. Without sciatica
    iii. Unsure
k. Bone stress injury
l. Asthma
m. Hypertension
n. Arrhythmia (e.g. atrial fibrillation, atrial flutter, supraventricular tachycardia)
o. Pelvic organ prolapse (feeling of a bulge into or out of the vagina)
p. Urinary incontinence
    i. Stress urinary incontinence (sudden leakage of urine during activities such as laughing, coughing, sneezing, or exercising)
    ii. Urgency urinary incontinence (sudden urge to urinate resulting in involuntary leakage of urine)
    iii. Combined
q. Fecal incontinence (unable to control bowel movements)
    i. Solid stool
    ii. Liquid Stool
r. Wind incontinence (unable to control gas)
s. Endometriosis
t. Polycystic Ovarian Syndrome (PCOS)
u. Amenorrhoea (I lost my period for 3 or more months other than due to previous pregnancy/postpartum, or contraception that stops period)
v. Relative Energy Deficiency in Sport (REDs) or Female Athlete Triad
w. Osteopenia
x. Osteoporosis
y. Sleep apnea
z. Insomnia
aa. Other
    i. Other medical conditions/symptoms. Please descibe

Pregnancy and Postpartum Health Outcomes

1. Did you experience any of the following medical conditions/symptoms DURING pregnancy AND/OR in the first 2 years FOLLOWING the pregnancy? Please check all that apply.

	DURING Pregnancy	3 months	6 months	12 months	24 months
No medical conditions	☐	☐	☐	☐	☐
Anxiety	☐	☐	☐	☐	☐
Depression	☐	☐	☐	☐	☐
Gestational diabetes (blood sugar outside of target ranges)	☐ ☐	☐ ☐	☐ ☐	☐ ☐	☐ ☐
Gestational hypertension (high blood pressure)	☐ ☐	☐ ☐	☐ ☐	☐ ☐	☐ ☐
Preeclampsia	☐	☐	☐	☐	☐

Hyperemesis gravidarium (extreme, persistent nausea and vomiting)	☐	☐	☐	☐	☐
Incompetent cervix/cervical insufficiency (weak cervical tissue)	☐	☐	☐	☐	☐
Intrauterine growth restriction (baby that is growing slowly)	☐	☐	☐	☐	☐
Placenta previa after 28 wks (placenta partially/completely covering cervix)	☐	☐	☐	☐	☐
Triplet or higher order multiple pregnancy	☐	☐	☐	☐	☐
Twin pregnancy	☐	☐	☐	☐	☐
Unexplained vaginal bleeding	☐	☐	☐	☐	☐
Pelvic girdle pain (pain in the back or front of the pelvis/groin and/or hips, either on one side or both)	☐	☐	☐	☐	☐
Low back pain	☐	☐	☐	☐	☐
Diastasis recti abdominus (abdominal bulging between the two abdominals along the midline)	☐	☐	☐	☐	☐
Pelvic organ prolapse (feeling of a bulge into or out of the vagina)	☐	☐	☐	☐	☐
Urgency urinary incontinence (feeling an urge to void and not reaching the toilet in time)	☐	☐	☐	☐	☐
Stress urinary incontinence (leaking when you jump, run, sneeze, cough)	☐	☐	☐	☐	☐
Combined urinary incontinence	☐	☐	☐	☐	☐
Fecal incontinence (unable to control bowel movements)	☐	☐	☐	☐	☐
Wind incontinence (unable to control gas)	☐	☐	☐	☐	☐
Premature labour (before 37 weeks)	☐	☐	☐	☐	☐
Sleep apnea (repetitive pauses, periods of shallow breathing while sleeping)	☐	☐	☐	☐	☐
Insomnia (hard to fall asleep or stay asleep)	☐	☐	☐	☐	☐
Carpal tunnel syndrome	☐	☐	☐	☐	☐
Other	☐	☐	☐	☐	☐

1. How much weight did you gain during pregnancy?
    a. Answer in pounds.
        i. OPEN BOX NUMBER VALIDATED
    b. Answer in kilograms.
        i. OPEN BOX NUMBER VALIDATED

Birth Outcomes

<u>For the rest of this survey</u>, we are interested in the MOST RECENT PREGNANCY.

1. How many times have you been pregnant (for any length of time)? (DROPDOWN NUMBERS)

2. How long since the end of your most recent pregnancy?
    a. I am currently pregnant.
    b. XXX months.
3. Did you undergo fertility treatments for this pregnancy?
    a. Yes
        i. Ovulation induction
        ii. Intrauterine insemination
        iii. In vitro fertilization
        iv. Intracytoplasmic sperm injection
        v. Other
            1. Open box
    b. No

4. Did this pregnancy result in a live birth?
    a. Yes
    b. No (NO BIRTH OUTCOMES)

5. During your most recent birth, what was the delivery mode?
    a. Vaginal, unassisted
    b. Vagnial, assisted (vacuum or forceps)
    c. Caesarean section, planned/elective/scheduled
    d. Caesarean section, emergency

6. During your most recent birth, did you experience perineal tearing?
    a. Yes
        i. 1st degree (small and skin deep, may or may not have required stitches)
        ii. 2nd degree (deeper and affect muscle of perineum)
        iii. 3rd degree (affect both muscle of perineum and anal sphincter)
        iv. 4th degree (further into linking of anus or bowel)
        v. Unsure
    b. No
7. What was your baby's sex? (ASK MULTIPLE TIMES FOR PARENTS OF TWINS/TRIPLETS)
    a. Female
    b. Male
    c. Prefer not to disclose
8. How many weeks pregnant were you when you delivered (your baby was born)? (DROPDOWN OF WEEKS)

9. How much did your baby weigh? (DROPDOWN OF WEIGHT FOR BOTH GRAMS AND LBS)

10. Did you experience any delivery complications

a. Postpartum hemorrhage
b. Infant shoulder dystocia
c. Other

11. Was your baby admitted to the Neonatal Intensive Care Unit (NICU)?
    a. Yes
        i. For how long?
    b. No

Breastfeeding

1. Did you breastfeed after childbirth (for any duration)?
    a. Yes
        i. exclusive breastfeeding
            1. when did you stop?
        ii. exclusive breastfeeding with supplemental pumping/expressing
            1. when did you stop?
    b. No
2. Did you have any baby related factors that impacted breastfeeding?
    a. Yes
        i. Lip tie
        ii. Tongue tie
        iii. Other
            1. Please specify
    b. No
3. Were there any work related factors that impacted your decision/ability to breastfeed?
    a. Yes
        i. Open box
    b. No

Prenatal Care Experiences

Thinking back to your most recent pregnancy, please answer the following questions about your prenatal care experiences.

2. Did you feel your needs were met by your healthcare provider(s) during pregnancy?
    a. Yes. Please explain why or how:
    b. Somewhat. Please explain why or how:
    c. No. Please explain why or how:

3. Did you feel satisfied with the prenatal care you received in your most recent pregnancy?
    a. Yes. Please explain why or how:
    b. Somewhat. Please explain why or how:
    c. No. Please explain why or how:

4. Did you feel your healthcare provider(s) were knowledgeable about your disability and the impact on your pregnancy?
    a. Yes
    b. No
    c. Somewhat

5. Ableism is considered discrimination and/or social prejudice on the basis of disability. Did you encounter any experiences of ableism during your prenatal care experiences?
    a. Yes. If comfortable, please describe:
    b. No

6. Is there anything you would like to add regarding your prenatal care experiences?

Training during Pregnancy

Research among people without physical disabilities recommends pregnant individuals engage in physical activity during pregnancy to benefit both the mother and child. However, there are currently no recommendations for physical activity during pregnancy among people with physical disabilities. This section will ask you questions about your experiences with physical activity during your pregnancy.

7. Was continuing to train and/or compete during pregnancy important to you?
    a. Yes. Please explain why:
    b. No. Please explain why:
    c. Somewhat. Please explain why:

8. Did you feel that continuing to train and/or compete during pregnancy was safe?
    a. Yes. Please explain why:
    b. No. Please explain why:
    c. I am not certain.

9. Were you told not to exercise, or be careful with exercising during your pregnancy?
    a. Yes
    b. No

10. Did anyone give you advice about being active during pregnancy?
    a. Yes.
        i. Who gave you this advice?
            1. Doctor
            2. Midwife or Doula
            3. Physiotherapist or Occupational therapist
            4. Exercise specialist
            5. Coach
            6. Friend or family
            7. Other, please describe:
        ii. What advice were you given?

b. No

11. Did you try to find resources about being physically active during your pregnancy?
    a. Yes
        i. Where did you look for resources? Select all that apply.
            1. Internet
            2. Healthcare provider
            3. Coach
            4. Books
            5. Other
    b. No

Engagement in sport during and after pregnancy.

The following questions will ask about your engagement in exercise and sport during pregnancy.

i. How long did you maintain your preconception level of training?

2. Did you make any modifications to your training during pregnancy (e.g. different equipment, reduced contact, change in type of training)?
    a. Yes
        i. Please describe any modifications that were made to your training during pregnancy (e.g. different equipment, reduced contact, change in type of training) and when/why they were made (weeks of pregnancy)? (OPEN BOX)
    b. No
3. Did you experience a sport-related injury during your pregnancy?
    a. Yes, please describe
    b. No

4. During pregnancy, did you train or compete under the following conditions?

During pregnancy, did you train or compete under the following conditions?	Yes or no	How often?	When did you stop this activity during pregnancy?	Did you have any health concerns as a result of this activity
Altitude above 1800m (5900ft) and below 2500m (8200ft)?	(RADIO BUTTONS)<br>- Yes<br>- No<br>- unsure	- 6x a week<br>- 5x a week<br>- 4x a week<br>- 3x a week<br>- 2x a week<br>- Once a week<br>- 3x a month<br>- 2x a month<br>- Once a month		- Yes<br>- No

		- Less than once a month		
Altitude above 2500m (8200ft)?	- Yes<br>- No<br>- Unsure			
Outdoor temperatures over 30 degrees Celsius (86 Fahrenheit) and below 35 degrees Celsius (95 Fahrenheit)?	(RADIO BUTTONS)<br>- Yes<br>- No<br>- Unsure			
Outdoor temperatures over 35 degrees Celsius (95 Fahrenheit)?	- Yes<br>- No<br>- Unsure			
Training above 90% of your maximum heart rate (For example near maximum effort)?	- Yes<br>- No<br>- Unsure			
Heavy weight training (For example lifting more than 80% of your pre-pregnancy body weight)?	- Yes<br>- No			
Exercise in a supine (laying on your back) position after 20 weeks of gestation?	- Yes<br>- No<br>- Unsure			

5. During pregnancy, did you experience any of the following?

During pregnancy, did you experience any of the following?	Yes or No	How often?	What activity were you doing when this happened?	How many weeks of pregnancy did this occur?	Did your health change after (e.g. injury, vaginal bleeding, miscarriage)?

Hard falls where you were concerned about the health of the fetus?	- Yes<br>- No				
Hard hits/tackles/collisions (either with by a person or an object (e.g. ball, stick)) where you were concerned about the health of the fetus?	- Yes<br>- No				

The following questions will ask about your engagement in exercise and sport following delivery.

1) How many weeks postpartum did you return to exercise (e.g., walking, cycling, swimming)? (DROPDOWN NUMBERS)

Type of activity	Returned postpartum	Weeks postpartum returned to activity	Why did you not return to activity? (ONLY IF NO RETURN)
High impact exercise (e.g., running)	• I returned to high impact exercise<br>• I did not return to high impact exercise<br>• I did not participate in high impact exercise prior to pregnancy		
**Training of contact sports (e.g., soccer/football, rugby)**	• I returned to contact/collision sports<br>• I did not return to contact/collision sports<br>• I did not participation in contact/collision sports prior to pregnancy		
**Full participation in your sport**	• I returned to training<br>• I did not return to my sport		

Competition	• I returned to competition<br>• I did not return to my sport		

2) Did you experience a sport-related injury during postpartum?
   a. Yes, please describe
   b. No

Level of Activity

When you think about your training during the pregnancy outlined above, how many types of activities did you do (e.g. Running, plyometrics, free-weight training =3).

1. These include: cardio, strength and sport specific training activities.
   a. Dropdown 1-6 – participants can select up to six from the following (other can be selected up to three times)
      1. Cycling – road
      2. Cycling – mountain
      3. Cycling – stationary
      4. Hand bike
      5. Jogging
      6. Rowing
      7. Running outdoors
      8. Running on a treadmill
      9. Running on an elliptical
      10. Swimming
      11. Wheelchair – ergometer
      12. Wheelchair – outdoors
      13. Other – open box
      14. Body weight resistance exercise
      15. Weight machines
      16. Free-weight training
      17. Olympic lifting
      18. Plyometrics
      19. Sport specific. Please specify. – open box
      20. Other. Please specify. – open box

Activity 1 Will be dropdown options and other options				
Drop down for activity	Open box if activity is 'other' or 'sport specific'	Number of sessions per week	Average duration (minutes per session)	Intensity (light, moderate or vigorous)

Activity 1 Will be dropdown options and other options				
Pre-Pregnancy	6 months			
During Pregnancy	1st Trimester (Weeks 1-13)			
	2nd Trimester (Weeks 14 – 27)			
	3rd Trimester (Weeks 28 – 40)			
Postpartum	0-6 weeks			
	6-12 weeks			
	12+ weeks			
